# Comparison of the Effect on Vessel Density and RNFL between Carteolol and Latanoprost

**DOI:** 10.3390/jcm11144159

**Published:** 2022-07-18

**Authors:** Elena Nutterova, Martin Fus, Libuse Bartosova, Iva Klimesova, Jan Lestak

**Affiliations:** 1 Ophthalmology Clinic JL, 155 00 Prague, Czech Republic; elenanutterova@gmail.com (E.N.); libuse.pileckova@email.cz (L.B.); 2 Faculty of Biomedical Engineering, Czech Technical University in Prague, 272 01 Kladno, Czech Republic; martin.fus@cvut.cz (M.F.); iva.klimesova@cvut.cz (I.K.)

**Keywords:** hypertensive glaucoma, OCTA, vessel density, RNFL, treatment with carteolol and latanoprost

## Abstract

The aim of the study was to compare the treatment of hypertensive glaucoma (HTG) in the early stages with carteolol and latanoprost by assessing the change in vessel density (VD) and retinal nerve fibre layer (RNFL). Methods: The first group with diagnosed HTG consisted of 46 eyes treated with carteolol; the second group consisted of 52 eyes treated with latanoprost. The following examinations were evaluated in all patients: intraocular pressure (IOP), retinal nerve fibre layer (RNFL), vessel density (VD) and visual field examination (glaucoma fast threshold test). The results were compared before treatment and 3 months after treatment. Results: There was no difference in the overall visual field defect (OD) between groups before treatment. After treatment, there was a decrease in IOP in both groups (carteolol-treated group had a mean decrease of 5.8 mmHg and latanoprost-treated eyes had a mean decrease of 7 mmHg). This difference was not statistically significant (*p* = 0.133). No similar difference was observed for RNFL (*p* = 0.161). In contrast, the change in the VD parameter was statistically significant between groups (*p* < 0.05), with a greater difference observed in the carteolol-treated group of eyes. Carteolol had a better effect on the VD.

## 1. Introduction

Hypertensive glaucoma (HTG), a neurodegenerative disease characterised by retinal ganglion cell (RGC) death and axon loss (RNFL), has a worldwide prevalence of approximately 3.5% in people aged 40 to 80 years. Due to the increasing number and proportion of older people in the population, it is assumed that 111.8 million people will have glaucoma by 2040. Currently available treatments cannot reverse glaucomatous damage to the visual system. However, early diagnosis and treatment can prevent the progression of the disease [1]. Most patients with HTG have no early symptoms. Conventional pharmacological glaucoma therapy and surgery to lower intraocular pressure (IOP) are not always sufficient; RGCs continue to die, and patients’ vision loss continues [2]. Therefore, slowing down the progression of the disease and preserving the quality of life are the main goals of HTG treatment. Lowering IOP is the only proven treatment for HTG. There is no dispute about the hypotensive effect of the antiglaucomatous drugs available today [3]. Due to the asymptomatic nature of the disease, treatment is often initiated too late and progression to severe visual impairment or blindness cannot be ruled out.

Conventional first-line treatment of glaucoma usually starts with the use of a topical selective or non-selective beta-blocker or a topical prostaglandin analogue [4]. The β-adrenoreceptor antagonist timolol and the prostaglandin F2α analogue latanoprost are two of the most important drugs used clinically to lower IOP in patients with glaucoma [5]. Prostaglandin analogues are recommended as the first-line treatment for HTG because of their efficacy, limited systemic side effects and once-daily dosing [6,7].

Latanoprost (Xalatan) is an ester analogue of prostaglandin F2α that reduces IOP by increasing uveoscleral outflow [8]. β-Blockers reduce intraocular fluid production by blocking β-adrenergic receptors in the ciliary epithelium. They are less effective during night-time hours because intraocular fluid production is naturally reduced at night. β-adrenergic blockers may have significant systemic adverse effects and are contraindicated in patients with a history of chronic obstructive pulmonary disease, asthma or bradycardia [9,10]. Ocular carteolol is a nonselective beta-adrenoceptor antagonist with intrinsic sympathomimetic activity. Ocular carteolol effectively reduces intraocular pressure in patients with HTG. Therefore, carteolol could protect the retina from light-induced damage with multiple effects, such as enhancing the antioxidative potential and decreasing the intracellular ROS production [11].

In our previous study, which compared the effect of prostaglandin and beta-blocker treatment in HTG using pattern defect (PD) and overall defect (OD), no difference between the evaluated parameters was found at 5-year follow-up. In the case of prostaglandins, PD showed no statistically significant changes (*p* = 0.35), but OD was close to a statistically significant difference (*p* = 0.09). For beta-blockers, we observed no changes in either PD (*p* = 0.37) or OD (*p* = 0.23) over the 5-year period [12].

Because visual field OD is more specific to HTG, it was surprising that this parameter approached statistical significance in eyes on prostaglandins [13]. As IOP increases, a number of biochemical and morphological processes occur in the visual pathway. Among the pathological processes, the most important is the damage of predominantly magnocellular retinal ganglion cells [14,15,16,17]. Changes in RNFL are likely to be secondary. Naskar et al. found in an experiment that up to 40% of retinal ganglion cells are lost within 2.5 months of glaucoma induction. Fundoscopic examination of the optic nerve head revealed cupping 2 months after induction of glaucoma [16]. In experimental glaucoma, retinal ganglion cell axons degenerate first in their retrolaminar and then in their intraocular part [18].

Because the magnocellular ganglion cells are located in the periphery of the retina [14,15,16,17] and are mostly undetectable in the early stages of HTG using the current perimetry tests (0–22, 0–30 degrees) [19], it was decided to observe the effect of treatment with latanoprost and carteolol on VD and RNFL before and after the 3-month treatment. For this selection, we were also guided by the findings of previous work, where we found a very strong correlation between VD and RNFL with increasing IOP. The correlation between IOP and OD was moderate [19].

## 2. Materials and Methods

This prospective cohort study was performed according to the Declaration of Helsinki and was approved by the internal ethics committee of the Ophthalmology Clinic JL (Prague, Czech Republic). All details, medical records, figures, medical history or test results were used with the written consent for publication from the patient, which are available from the corresponding author on reasonable request. All data used were anonymised.

**Figure 1:**
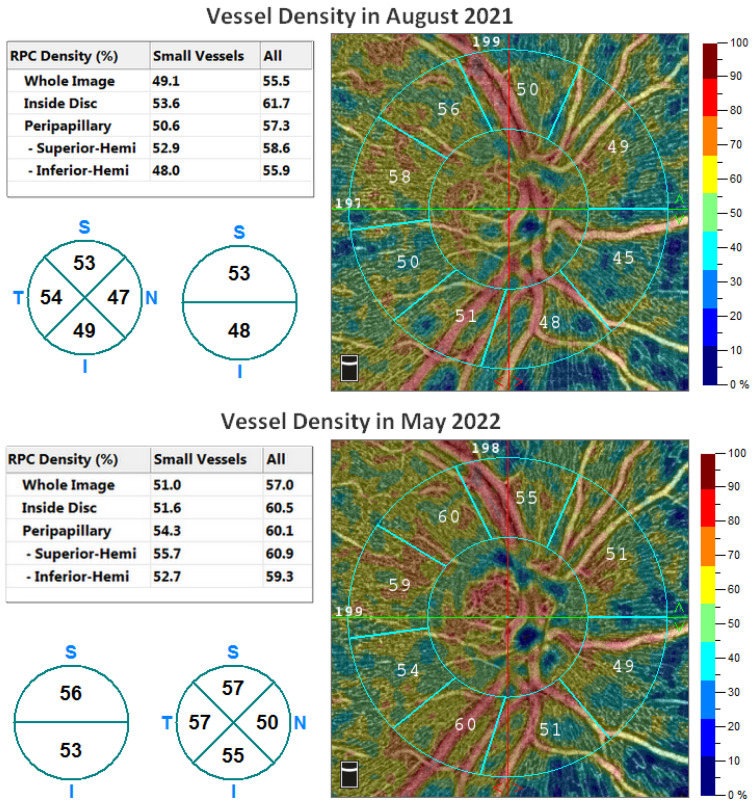
Comparison of the density of blood vessels in the patient’s right eye from October 2021 (upper part) and May 2022 (lower part).

Patients newly diagnosed with primary open-angle glaucoma (HTG), and with no previous antiglaucomatous treatment, were randomly divided into two groups with different medication. Inclusion criteria for the study: visual acuity 1.0 with possible correction of less than ±3 dioptres, approximately comparable changes in visual fields in all subjects, no other ocular or neurological diseases. The first group consisted of 46 eyes (total of 12 females and 11 males, mean age 47.3 years ±14.4) that were treated with carteolol (Carteol LP 2%) after diagnosis HTG. Patients without contraindications to the use of beta-blockers were included in this group. The second group consisted of 52 eyes (13 females and 13 males, mean age 56.4 ±13.6 years) that were treated with latanoprost (xalatan). IOP was measured non-contact with an Ocular Response Analyser (ORA) from Reichert Technologies, and the resulting IOP was averaged from the three measurements. RNFL thickness and vessel density were measured with the Avanti RTVue XR from Optovue. The visual field was examined with a fast threshold glaucoma program (the temporal part of the visual field up to 22 degrees and the nasal part up to 50 degrees), using a Medmont M 700 instrument (Medmont International Pty Ltd., Nunawading, VIC, Australia). The visual field overall defect (OD) parameter was used as evidence of approximately equally advanced HTG prior to treatment deployment and to categorise the patient cohort into groups (Table 1). The difference in VD and RNFL were compared before treatment and 3 months after deployment of the treatment. Vessel density assessment was further divided into the following parameters according to OCT angiography output: peripapillary vessel density of all vessels (PP-VDa), peripapillary vessel density of small vessels (PP-VDs), vessel density of all vessels of the whole image of OCT scan (WI-VDa) and vessel density of small vessels of the whole image (WI-VDs) of OCT scan (Figure 1). The measured values were subjected to statistical processing, and due to the age difference between the groups, statistical adjustment using multivariate regression models was necessary.

The Ethics Committee of the Ophthalmology Clinic of the JL FBMI CTU at its meeting on 11 January 2021 discussed the request of the Head of the Ophthalmology Clinic of the JL FBMI CTU about the intention to determine, in patients with different values of intraocular pressure, its influence on vessel densities, nerve fibre layer and visual field. The Commission found that this is not a clinical study, and the non-contact outpatient examinations performed will not affect or interfere with the health of the patient according to the Declaration of Helsinki of the World Medical Association (revised version 1 September 2000), and therefore this plan was not subject to approval by the Ethics Committee.

## 3. Results

Overall visual field defect (OD) before treatment was not statistically significant between the groups (*p* = 0.674), confirming approximately the same equal changes of glaucoma progression in both groups. The visual field examination after the initiation of antiglaucomatous treatment was not performed at identical time intervals and was therefore not evaluated. The mean measured values are shown in Table 1.

For the assessed parameter of RNFL change after treatment in both groups, the change was not statistically significant within each group (*p* > 0.05) or between the two groups (*p* = 0.161). A significant increase in VD was observed in eyes in the carteolol group. The differences were statistically significant (confidence interval 95%) when comparing the two groups and all vessel density parameters. The largest difference was in WI-VDa (*p* = 0.007). Thus, it can be concluded that latanoprost has a greater negative effect on small vessel density in the overall OCTA image.

## 4. Discussion

Visual field examination in HTG is the oldest of the above diagnostic methods and provides a picture of the entire visual analyser. Therefore, we selected patients with approximately the same changes in the visual field. Obviously, in the early stages of HTG, where the first changes occur in the magnocellular retinal ganglion cells, we cannot even theoretically detect a decrease in sensitivity in the central visual field [20]. The Medmont device uses a glaucoma program to examine the visual field, which examines the temporal part of the visual field up to 22 degrees and the nasal part up to 50 degrees. It is the examination of the nasal part that is very important in HTG. Gurcio and Allen found that the number of ganglion cells in the temporal part of the retina is three times smaller than in the nasal half [21]. Visual function assessment has always been an essential part of glaucoma diagnosis and monitoring, with static automated perimetry (SAP) being the gold standard 20 years ago. Glaucoma was first diagnosed on the basis of abnormalities in the visual field, but many patients have significant structural changes before changes are detectable on SAP. In addition, histological studies in humans and primates have shown that large numbers of retinal ganglion cells can be lost before statistically significant abnormalities are seen on SAP [22,23]. In a study on dead eyes, Kerrigan-Baumrind et al. estimated that a loss of at least 23–35% of RGCs is required for a statistically significant abnormality on SAP [22]. These studies suggest that reliance on SAP in early glaucoma is likely to lead to underestimation of the extent of glaucomatous damage. Harweth et al. take a similar view, concluding that RNFL thickness may be a more sensitive measurement for early stages, and SAP a more appropriate measurement for intermediate to advanced stages of glaucoma [24]. Visual field examination was performed only before the use of antiglaucomatous drugs. The reason for this was, in addition to the above-mentioned facts, the elimination of eyes with a possible other pathology in the visual pathway, but mainly the inclusion of eyes with the same changes in the visual fields (OD).

Because beta-blockers and prostaglandins are among the main drugs in monotherapy for HTG [25], we enrolled carteolol and latanoprost in our study. The fact that high IOP induced a significant decrease in vessel density per unit area in the laminar and retrolaminar regions of the optic nerve was described in their experimental work by Diaz et al. They also found a decrease in capillary length per unit volume. After the application of timolol and latanoprost, the diameter of the vessels of the studied area improved, but the capillary density did not change [26]. This conclusion is very important for early diagnosis and treatment of HTG.

The same authors investigated the loss of retinal ganglion cells in experimental glaucoma. In the groups treated with timolol, latanoprost, or brimonidine, the neuronal loss was less (331 ± 10, 360 ± 15, and 333 ± 3 cells/mm^2^, respectively), although values did not return to baseline levels in the control group (423 ± 11 cells/mm^2^) [27]. The issue of VD in glaucoma was also addressed by Liu et al. Reduction of IOP in prostaglandin-treated eyes compared with untreated eyes was significantly associated with an increase in papillary and peripapillary VD. This may indicate a mechanism by which IOP reduction modulates the risk of glaucoma progression by improving ocular microperfusion [28]. This may be one of the reasons why VD is altered before the degeneration of retinal ganglion cell nerve fibres. Conversely, with RNFL atrophy, there is a subsequent change in VD. This is also why VD testing in glaucoma is of such importance. This is confirmed by recent work [29,30,31,32,33,34,35,36].

Tamaki et al. measured the microcirculation in the optic nerve in the rabbit after carteolol treatment and demonstrated an improvement in blood flow [37]. They then performed similar measurements in glaucoma eyes and found improved blood flow after carteolol treatment in both rabbit and human eyes with glaucoma [38]. The reason for this will be not only the reduction in IOP, but also the blockage of beta-adrenergic receptors on small blood vessels [39]. There was an increase in VD after latanoprost application compared to the other eye where latanoprost was not applied [28]. The European Glaucoma Society’s latest 5th edition recommends prostaglandin analogues as the most effective drugs, and they are usually recommended as the first-choice treatment for HTG, also because of minimal systemic side effects [40]. As a result of our investigations in both groups, we found that both antiglaucomatous drugs had a significant effect on IOP reduction. We observed a greater decrease after latanoprost, although the difference between carteolol and latanoprost was not statistically significant. However, we see greater importance in the increase in VD, which was significant for both drugs. In eyes on carteolol, the increase in VD was greater and, compared to latanoprost, this difference was statistically significant. We have not seen similar work in the available literature and believe that this will assist ophthalmologists in their decision making regarding the indication of antiglaucoma treatment. OCT angiography is a rapidly developing technology whose role in glaucoma treatment is not defined [40]. Our study also shows that the importance of OCT angiography in the diagnosis and management of hypertensive glaucoma is significant.

## 5. Conclusions

Comparison of both antiglaucomatous drugs showed a better effect on vessel density with carteolol compared to latanoprost.

## Figures and Tables

**Table 1:** Summary data of analysis.

	Carteol		Xalatan		*p*-Value
	Mean	SD	Mean	SD	
**count**	46		52		X
**age**	47.260	14.411	56.38	13.56	0.002
**OD**	2.090	1.194	2.197	1.314	0.674
**diff PP-VDa (%)**	1.384	2.748	0.217	2.157	0.021
**diff PP-VDs (%)**	1.378	1.930	0.306	2.264	0.014
**diff WI-VDa (%)**	1.170	1.505	0.150	2.059	0.007
**diff WI-VDs (%)**	1.102	2.224	0.148	2.044	0.029
**diff RNFL (um)**	0.065	3.586	0.885	2.036	0.161
**diff IOP (mmHg)**	−5.780	3.794	−7.008	4.173	0.133

PP-VDa (peripapillary vessel density of all vessels), PP-VDs (peripapillary vessel density of small vessels), WI-VDa (vessel density of all vessels of the whole image), WI-VDs (vessel density of small vessels of the whole image), RNFL (retinal nerve fibre layer), OD (overall defect), IOP (intraocular pressure), mean (mean value), SD (standard deviation), *p*-value (significance testing parameter). Mean column for VD, RNFL and IOP shows the diff (difference) against pre-treatment values.

## Data Availability

Data are available on personal request from the corresponding author.
